# Role of PCE Anionicity in Early Hydration and Setting Behavior of NaOH-Activated Slag Binder

**DOI:** 10.1021/acsomega.6c02392

**Published:** 2026-06-19

**Authors:** Hsien-Keng Chan, Li-Chia Chang, Zuiliang Deng, Johann Plank, Ana Paula Kirchheim, Yue Zhang, Lei Lei

**Affiliations:** † 9184 Technische Universität München, Construction Chemistry, Lichtenbergstraße 4, Garching bei München 85747, Germany; ‡ Akzo Nobel Swire Paints (Shanghai) Ltd., No. 536 Rong Le Road E, Song Jiang Industrial Zone, Song Jiang District, Shanghai 201600, China; § Shanghai Sunrise Polymer Material Co., Ltd., 3F Bldg. 3 No. 188 Pingfu Rd., Juxin Park-Caohejing Hitech Park, Xuhui District, Shanghai 200231, China; ∥ 28124 Universidade Federal do Rio Grande do Sul (NORIE/UFRGS), Department of Civil Engineering, Post-Graduate Program in Civil Engineering: Construction and Infrastructure, Av. Osvaldo Aranha 99 (Engineering Campus—Central Area), Porto Alegre, Rio Grande do Sul 90035-190, Brazil; ⊥ Xi’an Institute of Hi-Tech, Zhijian Lab, Xi’an, Shaanxi 710025, China; # Hunan University, Key Laboratory for Green & Advanced Civil Engineering Materials and Application Technology of Hunan Province, College of Civil Engineering, No. 2 Lushan South Rd, Yuelu District, Changsha 410082, China

## Abstract

Several studies have revealed that polycarboxylate superplasticizers (PCEs) with shorter side chains and higher anionicities can overcome challenges posed by the high alkaline environment and disperse alkali-activated slags (AASs). However, how adsorption–dispersion translates into macroscopic setting behavior and early hydration remains insufficiently understood. Here, a series of isoprenyl oxy poly­(ethylene glycol) ether (TPEG) PCEs were synthesized with an identical side-chain length (*n*_EO_ = 10) but different anionicities by varying the feeding molar ratio of acrylic acid (AA:TPEG = 3, 4.5, and 7), enabling the isolated investigation of anionicity effects. A comprehensive analysis was conducted on NaOH-activated slag using adsorption measurements, ζ-potential analysis, isothermal calorimetry, setting time determination, compressive strength testing, and XRD characterization. It was found that an increase in PCE anionicity could enhance adsorption by stronger Ca^2+^ complexation, providing not only better dispersion but also a longer setting time and slightly delayed and reduced heat flow peak. However, these drawbacks did not compromise early strength development because the high-anionicity PCE can promote better particle stabilization. It was proposed that the more effectively dispersed the system is, the more prolonged the dissolution process may become, leading to delayed local precipitation. Consequently, hydration progression may be sustained, resulting in a higher compressive strength. These findings provide insight into the role of polymer charge density in influencing hydration and strength evolution in the NaOH-activated slag.

## Graphical abstract

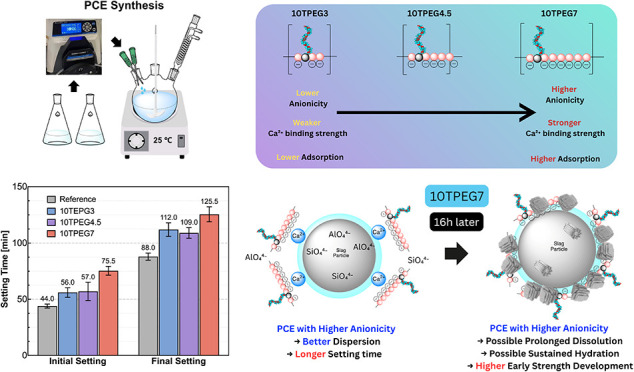

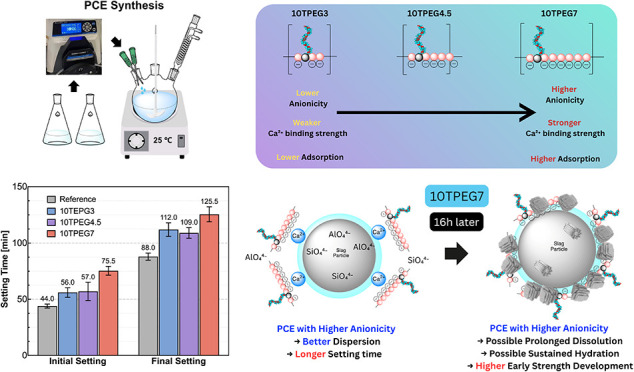

## 1 Introduction

Ordinary Portland cement (OPC) is a vital binder for the construction industry; however, its production can generate substantial CO_2_ emissions. In fact, Josa^1^ demonstrated that about 0.7–1.0 tons of CO_2_ were released into the atmosphere for every ton of cement manufactured. This massive amount of CO_2_ emissions can only worsen the anthropogenic catastrophe of global warming.^2^ Therefore, for years, researchers have studied different methods to mitigate CO_2_ emissions from the construction sector. Several greenhouse gas (GHG) reduction strategies have been proposed in this sector, including thermally efficient kiln preheaters,^3^ renewable or biomass-derived fuels,^4^ novel grinding aids,^5^ carbon capture and storage (CCS),^6^ and clinker substitution.^7^ The latter strategy has been identified as one of the most effective ways, since partially or fully substituting clinker with supplementary cementitious materials (SCMs) can not only resolve the decarbonization issue in the cement sector but also provide a utilization solution for the byproducts of other sectors. One possible alternative is ground granulated blast furnace slag (GGBFS).^8,9^

GGBFS is a byproduct of the iron and steel industry; unlike OPC, pure GGBFS exhibits latent hydraulic properties,^10^ which require activation, such as alkali-activated slag (AAS). The GGBFS is composed primarily of calcium oxide (CaO), silicon dioxide (SiO_2_), aluminum oxide (Al_2_O_3_), magnesium oxide (MgO), and other alkali metals such as Na_2_O.^11^ As a vitreous material rich in silicates, it dissolves readily under alkaline conditions, releasing Ca^2+^, Si^4+^, and Al^3+^ ions.^12^ Owing to its latent hydraulic and pozzolanic properties, GGBFS has been widely employed in several cementitious systems, including supersulfated cements,^13^ alkali-activated binders,^11,14–17^ and ordinary Portland cement (OPC) with high levels of GGBFS substitution.^14,18–21^ The use of GGBFS as a partial or complete substitute for OPC offers the following advantages: (a) a great way to utilize solid waste (GGBFS): in fact, the percentage reduction in CO_2_ emissions from OPC due to clinker replacement is estimated to range from 55 to 75%;^22^ (b) higher compressive strength: it was reported that the mechanical property from the water-glass-activated slag system is better than pure OPC mortar;^23^ and (c) superior chemical degradation resistance from AAS concretes compared to OPC-based concretes, as demonstrated by their performance in environments with elevated sulfate, chloride, or acid concentrations.^24^ Such a high-pH environment caused by these alkalis has two primary effects: it first destabilizes the silicate network and second, it accelerates the dissolution of slag particles, thereby speeding up the nucleation and precipitation of calcium-silicate-hydrate (C-S-H) and calcium-alumino-silicate hydrate (C-A-S-H) gels. The generation of both C-S-H and C-A-S-H gels is contingent upon the release of an increasing quantity of calcium (Ca^2+^), silicon (SiO_4_^4–^), and aluminum (AlO_4_^4–^) ions into the pore solution.^25^ This ultimately hastens the hydration of the GGBFS, leading to the so-called alkali-activated slag, AAS.

Typically, AAS exhibits inferior fresh properties compared to OPC in practical applications.^26^ Moreover, conventional water-reducing admixtures (WRAs), particularly polycarboxylate ether (PCE)-based superplasticizers, often do not perform well in such systems. The admixtures need to overcome the following challenges in order to improve both fresh and mechanical properties of the AAS: (a) insufficient availability of calcium;^27^ (b) competition between PCE and anions for adsorption sites;^28^ (c) solubility issues;^29^ and (d) performance degradation^30–32^ caused by the high ionic strength arising from both chemical compositions of the binder and activator. PCEs, which provide dispersion through both electrostatic repulsion and steric hindrance,^33,34^ could resolve the lower workability and rapid setting time issues of AAS since the structure of these comb polymers can be easily modified, enabling researchers to design a range of desired molecular structures suitable for the AAS system. Conte et al.^35^ found that α-allyl-ω-methoxy poly­(ethylene oxide) ether (APEG)-type PCE with maleic anhydride (MA) as the backbone monomer could disperse the NaOH-activated slag due to the strong chelating ability between maleic anhydride and free Ca^2+^ cations.

In contrast, Lei and Chan^36^ introduced another type of PCE, the α-methallyl poly­(ethylene glycol) ether (HPEG)-type PCE, and demonstrated that this polymer, with shorter side chains, higher molecular weight, and higher anionicity, provides the best dispersion. Li et al.^37^ then compared the dispersing capacity of APEG- and HPEG-based PCE synthesized with acrylic acid (AA) in the AAS system and discovered that the HPEG-based polymer offers better fluidity since it offers more AAA and AAE (A: acid and E: ethers) polymer sequences, which enhance its adsorption. Additionally, Li et al. further confirmed Lei et al.’s finding on providing direct evidence that PCE with a higher *M*_w_ could generate a higher adsorbed layer thickness (ALT) since it can be adsorbed as the “tail” conformation.^38^ Although the scarcity of free Ca^2+^ in the pore solution of AAS had been previously noted, two studies by Zhang et al. showed that this limitation, alongside solubility constraints, is one of the primary causes underlying the ineffectiveness of conventional PCEs in dispersing this green binder. Their studies have revealed that (a) the backbone monomer plays a decisive role, with acrylic acid (AA) exhibiting better compatibility due to stronger Ca^2+^ chelation than methacrylic acid (MAA)^39^ and (b) supplementing calcium salts can enhance the PCE’s adsorption and improve fluidity in the other two activated systems (Na_2_CO_3_ and Na_2_SiO_3_), which are known for not being easily dispersed.^40^

While extensive studies have shed light on the desired structures of PCEs that can improve the workability of AAS pastes and the adsorption efficiency, the coupling between PCE anionicities and early hydration, e.g., setting time behavior and subsequent strength development, remains insufficiently understood. As a result, these early-age reaction behaviors are two of the remaining challenges hindering the real construction applications of such a green binder system. Hydration kinetic characteristics also play an essential role in engineers’ considerations when implementing alkali-activated materials (AAMs) in industrial-scale applications. Thus, a better understanding of how PCEs influence the setting behavior of AAS could help transform this green binder system from a specialty binder into a versatile ‘workhorse’ binder, thereby broadening its applications beyond precast concrete to ready-mix concrete and shotcrete. Rapid setting is problematic since it directly affects casting, finishing, and structural performance timelines. Conversely, slow compressive strength development may fail to meet construction standards. However, there are still insufficient studies in the literature discussing the impact of PCE anionicity on hydration kinetics. For instance, da Silva et al.^41^ reviewed over 100 publications and found only one that explicitly discussed how superplasticizers could affect the setting time of AAS, highlighting the research niche in this crucial area. Similarly, Palacios and Puertas^30^ investigated the effect of ω-methoxy poly­(ethylene glycol) methacrylate (MPEG)-type PCE in a 4% by weight of slag (bwos) NaOH-activated slag system and found that MPEG-PCE slightly increased the initial setting time and slightly improved the compressive strength of AAS mortar. However, beyond this isolated finding, there is a notable lack of systematic studies investigating how variations in PCE molecular design affect these key properties in AAS.

To address this gap, PCEs with different anionicities were synthesized from isoprenyl oxy-polyethylene glycol ether (TPEG) and acrylic acid. Different anionicities were achieved by adjusting the feeding molar ratios of acrylic acid to macromonomer (3, 4.5, and 7). Moreover, all polymers possessed a short side-chain length (number of ethylene oxide units, *n*_EO_ = 10). This side-chain length was intentionally selected not only because polymers with shorter side chains generally provide enhanced dispersion and solubility in alkaline pore solutions but also because the reduced steric hindrance effect (compared to PCEs with *n*_EO_ = 23) allows the effects of charge density and adsorption-related interactions to be more clearly isolated. Based on this design, the present study investigates how these PCEs with different anionicities affect the setting time and hydration kinetics of a 4% by weight of slag (bwos) NaOH-activated slag system. This systematic design establishes direct relationships among the polymer structure, adsorption characteristics, hydration kinetics, setting behavior, and compressive strength development in NaOH-activated slag systems. The findings are expected to provide insights into structure–property relationships, and, ultimately, offer molecular-level design guidance for next-generation PCEs to achieve a balanced performance in workability, setting control, and early-age performance. Finally, this could support the broader implementation of low-carbon alkali-activated binders in practical construction applications.

## 2 Materials and Methods

### 2.1 Materials

#### 2.1.1 Chemicals

Acrylic acid (AA, >99% purity, provided by Shanghai Sunrise Co. Ltd., China), isoprenyl oxy-polyethylene glycol ether macromonomer (TPEG, >98% purity, obtained from Shanghai Dongda Chemical Co. Ltd., China, with an average EO unit number of 10), 2-mercapto-ethanol (β-Me, >99% purity, manufactured by Sinopharm Chemical Reagent, China), and 32 wt % sodium hydroxide solution (obtained from Nanjing Regu Co. Ltd., China) were all used without further purification.

#### 2.1.2 Slag

Ground granulated blast furnace slag (GGBFS) was obtained from Shanghai Huanyu Construction Materials Co. Ltd., China. It complied with the Chinese standard GB/T 18046-2008, classified as S95 slag (fineness ≥ 400 m^2^/kg). Table 1 lists the oxide composition of the slag sample determined by X-ray fluorescence analysis (XRF).

**Table 1:** Oxide Composition of the Slag Used in This Paper

Oxide	[wt %]
CaO	50.6
SiO_2_	24.3
Al_2_O_3_	15.4
MgO	5.1
SO_3_	1.6
TiO_2_	0.8
Fe_2_O_3_	0.5
MnO	0.4
K_2_O	0.3
Na_2_O	0.3
BaO	0.3
SrO	0.2
ZrO_2_	0.1
Total	99.9

### 2.2 PCEs Synthesis and Characterization

#### 2.2.1 Preparation of TPEG Copolymers

PCEs with different amounts of anionic monomer were synthesized via free-radical copolymerization, as shown in Figure 1, and the designed molar ratios of AA:TPEG are enumerated in Table 2. The synthesis procedure of 10TPEG3 (made from AA:TPEG at a molar ratio of 3 and a side chain of 10 EO units) as an example is shown as follows: a 1 L four-necked round-bottom flask connected with a thermometer, a mechanical stirrer, and two separated feeding inlets was charged with 150.00 g (250 mmol) of TPEG and 8.97 g (124 mmol) of AA, which later dissolve with 275.85 g of DI water. Next, two solutions were prepared. Solution A contained 1.37 g (7.78 mmol) of l-ascorbic acid and 21.20 g of DI water, while Solution B consisted of 1.98 g (19.2 mmol) of 2-mercaptoethanol (βME), 45.07 g (25.34 mmol) of AA, and 30.53 g of DI water. Solutions A and B were added separately to the three-necked round flask using peristaltic pumps for 40 min after the addition of 5.50 g (0.162 mmol) of H_2_O_2,_ prior to the start of the reaction. After the addition of the two solutions, the reaction mixture was stirred at room temperature for an additional 30 min, for a total reaction time of 70 min. After the addition of the two solutions, the polymer’s pH was adjusted to 6.5–7 with 32% NaOH, yielding a PCE solution with a solid content of ∼35%.

**Figure 1:**
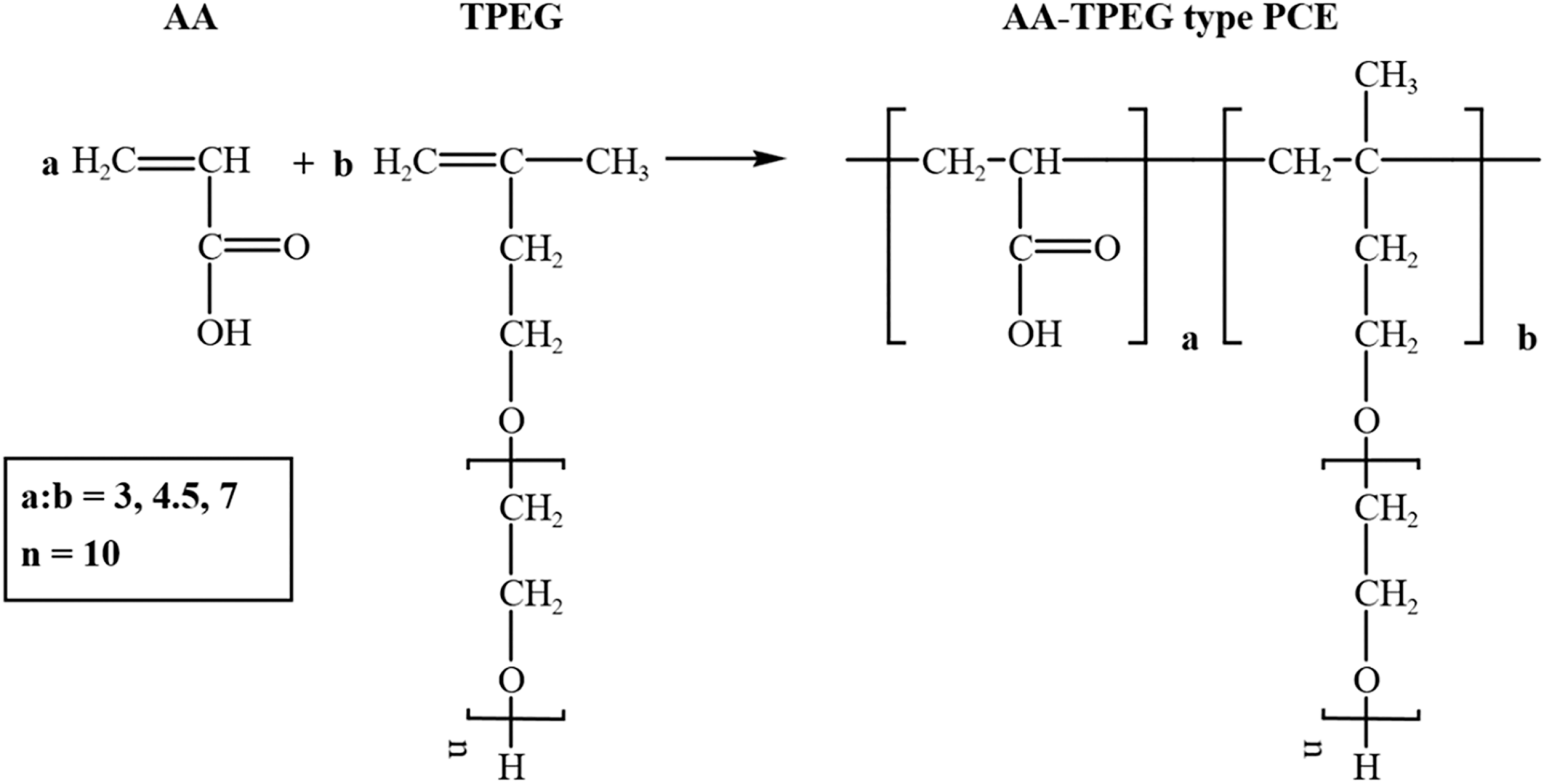
Reaction scheme for the synthesis of TPEG-based PCE via free-radical polymerization.

**Table 2:** Structural Composition of PCE Samples and AA:TPEG Feed Molar Ratios

Structural composition of PCE	Ethylene oxide unit of TPEG	Molar ratio of AA:TPEG
10TPEG3	10	3.0:1
10TPEG4.5	10	4.5:1
10TPEG7	10	7.0:1

#### 2.2.2 Size Exclusion Chromatography (SEC)

The conversion, the average molecular weight, and the polydispersity index of copolymers were determined by *dn/dc* value of 0.135 mL/g (value for PEO)^42^ with gel permeation chromatography (GPC) using a Water Alliance 2695 Separation Module (Waters, Eschborn, Germany) equipped with three Ultrahydrogel columns (120, 250, 500) and an Ultrahydrogel Guard column. The samples were analyzed using a 0.1 M NaNO_3_ solution as the eluent at a flow rate of 0.5 mL/min. The GPC samples (10 g/L) were prepared by diluting the PCE solution with 0.1 M NaNO_3_ (pH = 12) and filtered through a 0.22 μm syringe filter before measurement.

#### 2.2.3 Anionic Charge Measurement

The anionic charge density of the PCE polymer was measured using a particle charge detector (PCD 03 pH, Mütek Analytic, Germany). PCE solution was prepared at a concentration of 0.5 g/L in a 100 mL volumetric flask either in DI water or 0.1 M NaOH (pH of 13). Then, 10 mL of PCE solution was transferred to the particle charge detector and its anionic charge was determined by titration with polyDADMAC solution (0.34 g/L) until the streaming potential reached zero. Each sample was measured 3–5 times, and the average value was reported as the anionic charge density.

### 2.3 Experiments with AAS Paste

#### 2.3.1 Dispersing Performance of PCEs in AAS Paste

The workability of AAS paste with various TPEG-based PCEs was determined using the modified “mini-slump” test according to DIN EN 1015 and GB/T8077-2008. First, the water-to-slag ratio (w/s ratio) was set at 0.6, as at this w/s ratio the paste spread flow was 18 ± 0.5 cm without any admixture, e.g., PCE. In a typical experiment, 300 g of slag was added to a porcelain cup containing 37.5 g of 32% NaOH (activator dosage of 4% by weight of slag), along with the required amount of water and PCEs. The mixture was then mixed with a blender (NJ-160A, WuXi Jianyi Instrument & Machinery Co. Ltd., China) at a slow speed (140 rpm) for 120 s, followed by a 15 s pause and another 120 s of stirring at a fast speed (285 rpm). Before the measurement, the mixture was stirred manually to prevent sedimentation and poured into a Vicat cone (height 60 mm, top diameter 36 mm, bottom diameter 60 mm) on a glass plate. Once the paste was filled to the rim, the cone was immediately lifted vertically. The vertical and horizontal diameters of the paste were measured after it stopped spreading, and the average of the two diameters was taken as the spread flow.

#### 2.3.2 Setting Time Measurement

The setting time was measured using an automatic Vicat apparatus according to DIN EN 196-3 and Chinese standard GB/T 1346-2011. The AAS paste was prepared in the same way as in Section 2.3.1, but with a fixed PCE dosage of 0.3% bwos and a w/s ratio of 0.4. The slag paste was first poured into a Vicat mold placed on a plastic base plate. After a scraper was used to ensure a smooth surface on the top, the initial and final setting times were tested. The initial setting time was marked when the distance between the needle and the base plate was 4 mm. At each 15 min test interval, the final setting time was recorded when the ring attachment no longer marked the specimen. The entire experiment was conducted in a curing room at 20 ± 1 °C and 90% relative humidity (RH). Both the initial setting time and the final setting time were measured at least three times for each sample, and the average values were reported.

#### 2.3.3 Isothermal Heat Flow Calorimetry

The hydration kinetics of the AAS paste with PCEs were monitored using an isothermal heat-conduction calorimeter (TAMair, Thermometric, Järfälla, Sweden). In this experiment, 4 g of slag was filled into 10 mL glass ampules and mixed with a solution containing the mixing water (w/s ratio of 0.6), the specified dosage of PCEs (the dosage at which the spread flow could reach 26 cm in the mini-slump test, 0.21, 0.23 and 0.43% bwos for 10TPEG7, 10TPEG4.5 and 10TPEG3, respectively), and 0.16 g of NaOH (4% bwos). The vials were then vortex-shaken for 2 min and immediately transferred to the calorimeter. Heat flow curves were recorded for approximately 3 days until heat release ceased.

#### 2.3.4 X-ray Diffraction (XRD)

The hydrate products of AAS paste mixed with PCE polymers were observed using an X-ray diffractometer (Bruker D8 Advance, Germany). Samples were mounted on the sample holders using backloading to minimize preferred orientation. Scans were recorded at a 2θ range of 4–75° with Cu Kα radiation at 30 kV and 35 mA, using a step size of 0.015° and a total measuring time of 78.9 min per scan. For sample preparation, the slag paste was prepared as in Section 2.3.1, using the same PCE dosages as in Section 2.3.5. The paste was then poured into a 1 × 1 × 1 cm mold. The mold was then placed in the climate chamber (20 ± 1 °C and 90% RH). After curing for 1 day, the samples were polished with 300-, 600-, and 1000-mesh sandpaper and tested.

#### 2.3.5 Total Organic Carbon (TOC)

The adsorption of PCEs onto AAS was determined by TOC analysis. The same w/s ratio and the activator dosage were applied in accordance with Section 2.3.1. A certain amount of PCE solution, DI water, and 0.2 g of NaOH (4% bwos) were charged into a 15 mL centrifuge tube. Then, 5 g of slag was weighed and transferred into the centrifuge tubes. Later, the mixture was shaken by a wobbler for 2 min. After these centrifuge tubes were centrifuged at 8500 rpm for 5 min, the upper supernatant was extracted with a syringe and filtered through a 0.2 μm poly­(ether sulfone) syringe filter (Model FPS250020, Graphic Controls, New York, USA). Then, these filtered solutions were diluted with DI water and the pH was adjusted to ∼2 with 0.1 M HCl. Finally, TOC measurements were performed using a TOC-L Series (Shimadzu Corporation, Japan). Each prepared TOC sample was measured at least twice. Additional measurements were conducted if large deviations were observed, and the mean values were reported.

#### 2.3.6 ζ-Potential Measurement

The ζ-potential of PCEs on the slag particle surface was measured using a DT 1200 Electroacoustic Spectrometer manufactured by Dispersion Technology, Inc., Bedford Hills, NY, USA. The w/s ratio was set at 0.6. The experimental conditions were consistent with those in Section 2.3.1, except that PCE polymers were added gradually. After the paste was prepared, it was transferred to a container. The container was then placed in a ζ-potential instrument and stirred continuously at 200 rpm. The ζ-potential of the slag particles was measured with a stepwise titration of PCE solution.

### 2.4 Experiments with AAS Mortar

#### 2.4.1 Dispersing Performance of PCEs in Mortar Test

The dispersion efficiency of the PCE samples was measured at a water-to-slag ratio of 0.5 and a binder-to-sand ratio of 1:3. Mixing was carried out according to DIN EN 196 and GB/T 8077-2000. With a JJ-5 eccentric agitator (WuXi Jianyi Instrument & Machinery Co. Ltd., China), the mortar was prepared as follows: first, 450 g of slag was mixed with 0.82 g of DFP04 defoamer powder (supplied by Shanghai Sunrise Co. Ltd., China) by dry blending in a bag. Then, 56.25 g of 32% NaOH (activator dosage 4%, bwos) and the required amount of PCEs and water were added to the mixing bowl. Once the agitator started mixing at 140 rpm for 30 s, 1350 g of standard sand was automatically added. Upon completion of the standard sand addition, the mixing speed was increased to 285 rpm for 30 s, followed by a 90 s pause, and then resumed mixing at 285 rpm for 60 s. During the 90-s interval, a scraper was used to push the mixture back into the bowl along its edges, and the mortar was manually stirred for at least 10 s to prevent sedimentation.

The flowability of the mortar was determined according to DIN EN 1015-3, and the mortar was first scooped into a two-layer mold (height 40 mm, top diameter 70 mm, bottom diameter 80 mm) placed at the center of the wet metal plate. Each layer was tamped 10 times with a tamp rod. Then, the upper mold was lifted vertically, and the mortar was leveled with the upper edge of the lower mold. Any excess mortar that fell on the tabletop was removed. The bottom mold was then immediately removed by steadily lifting it and then dropping it 15 times at 40.0 mm in 15 s. When the mortar stopped flowing, the vertical and horizontal diameters were measured and averaged to obtain the spread flow value.

#### 2.4.2 Mortar Compressive Strength

The compressive strength of the AAS mortar was measured according to the standard DIN EN 196-1 after 8 h, 16 h, 1 d, 3 d, 7 d and 28 d. The mortar was prepared as described in Section 2.4.1 (w/s = 0.5, binder:sand ratio of 1:3). The PCE dosages were 0.33, 0.48, and 1% bwos for 10TPEG7, 10TPEG4.5, and 10TPEG3, respectively. The mortar was poured into 40 × 40 × 160 mm molds and oscillated 60 times over 60 s to remove air. The molds were then placed in a curing room at 20 ± 1 °C and 90% RH. The mortar specimens were first demolded from the molds and then tested for compressive strength. For specimens with more than 1 d strength tests, samples were demolded and cured in the climate chamber (20 ± 1 °C and 90% RH). For each sample, a total of six specimens were tested for compressive strength at each curing age, and the average values were reported.

## 3 Results and Discussion

### 3.1 Characteristic Properties of PCE Samples

All of the TPEG-based PCEs were successfully synthesized. A representative GPC spectrum of 10TPEG7, as illustrated in Figure 2, exhibits both light scattering (LS) and differential refractive index (dRI) signals. In this dRI spectrum, the initial peak with a brief retention time corresponded to the polymer fraction present in the sample, while the subsequent peaks with longer retention times reflected the residual monomer and the solvent peak.

**Figure 2:**
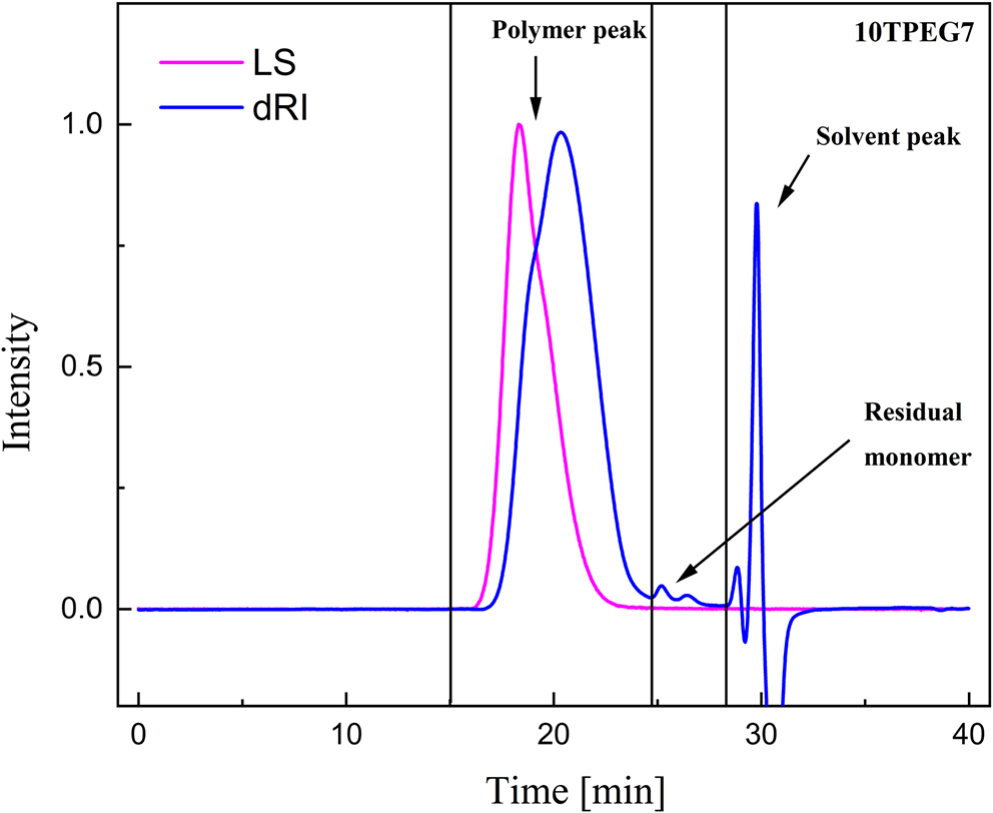
GPC spectrum of 10TPEG7.

Table 3 presents the polymer yield, the averaged molecular weights (*M*_w_ and *M*_*n*_), the polymer dispersity index (PDI), and the conversion of the synthesized copolymers as determined by GPC. All PCEs exhibited high quality, as evidenced by high macromonomer conversion (>90%) and relatively low polydispersity indices (PDI < 2.5). Moreover, all three PCEs have a relatively similar molecular weight (*M*_w_) ranging from 31,000 to 42,000 Da.

**Table 3:** Characteristic Molecular Parameters of Synthesized TPEG-Based PCE Polymers

PCE samples	*M*_w_ [Da]	*M*_*n*_ [Da]	PDI [*M*_w_/*M*_*n*_]	Macromonomer conversion [%]
10TPEG3	31,000	14,000	2.2	92
10TPEG4.5	42,000	19,000	2.2	90
10TPEG7	35,000	26,000	1.4	95

The anionicity of the synthesized PCEs was determined by particle charge detection with two selected conditions: (i) 0.1 M NaOH (pH = 13) and (ii) 0.1 M NaOH containing 1 g/L Ca^2+^.^43^ The former simulates the high pH of the AAS’s pore solution, while the latter evaluates the PCEs’ Ca^2+^ chelation ability, since several studies have pointed out that the calcium-binding capacity could be an essential parameter.^35,40^ As shown in Figure 3, increasing the feeding molar ratio of AA:TPEG leads to an increase in the anionicity under alkaline conditions, but vice versa in the case of 1 g/L Ca^2+^. This inverse trend suggests that 10TPEG7 is likely to be adsorbed onto slag particles more readily, due to its higher calcium affinity than the other two PCE samples.

**Figure 3:**
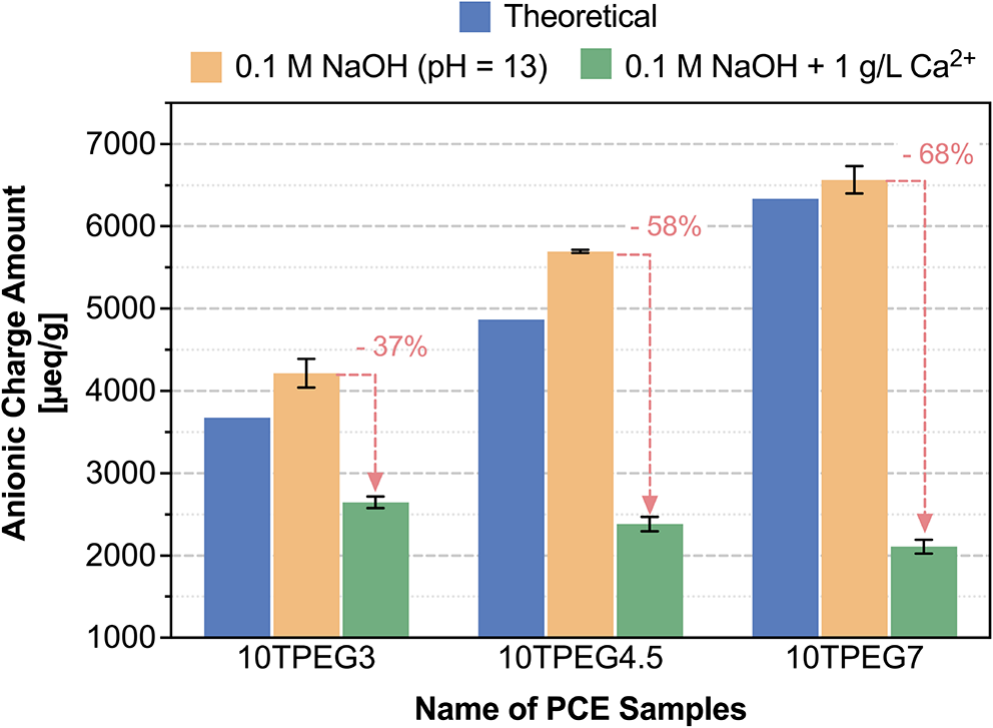
Anionic charge density of synthesized TPEG-PCE polymers.

### 3.2 Influence of PCEs on Fluidity of AAS

Mini-slump tests were conducted to investigate the dispersing ability of the synthesized PCEs. The requisite dosages of PCEs with varying anionicities were determined to achieve a targeted spread flow of 26 cm in AAS paste. The results are presented in Figure 4. It can be observed that the copolymer with higher anionicity (a higher molar ratio of AA:TPEG) requires a lower dosage to achieve the desired spread flow of 26 ± 0.5 cm, as expected. This result corroborates the findings of previous studies. For example, Lei and Chan reported that HPEG-based PCEs with longer side chains but higher anionic charge can disperse the AAS paste.^36^ In fact, for 10TPEG3, the required dosage is twice that of 10TPEG7 (0.43 vs 0.21% bwos) to achieve the same fluidity. One potential explanation is that copolymers with higher anionicity exhibit enhanced adsorption capacity, necessitating lower dosages to achieve optimal dispersion. To substantiate this hypothesis, ζ-potential and TOC measurements were conducted to ascertain the impact of anionicity on the dispersing performance of PCE.

**Figure 4:**
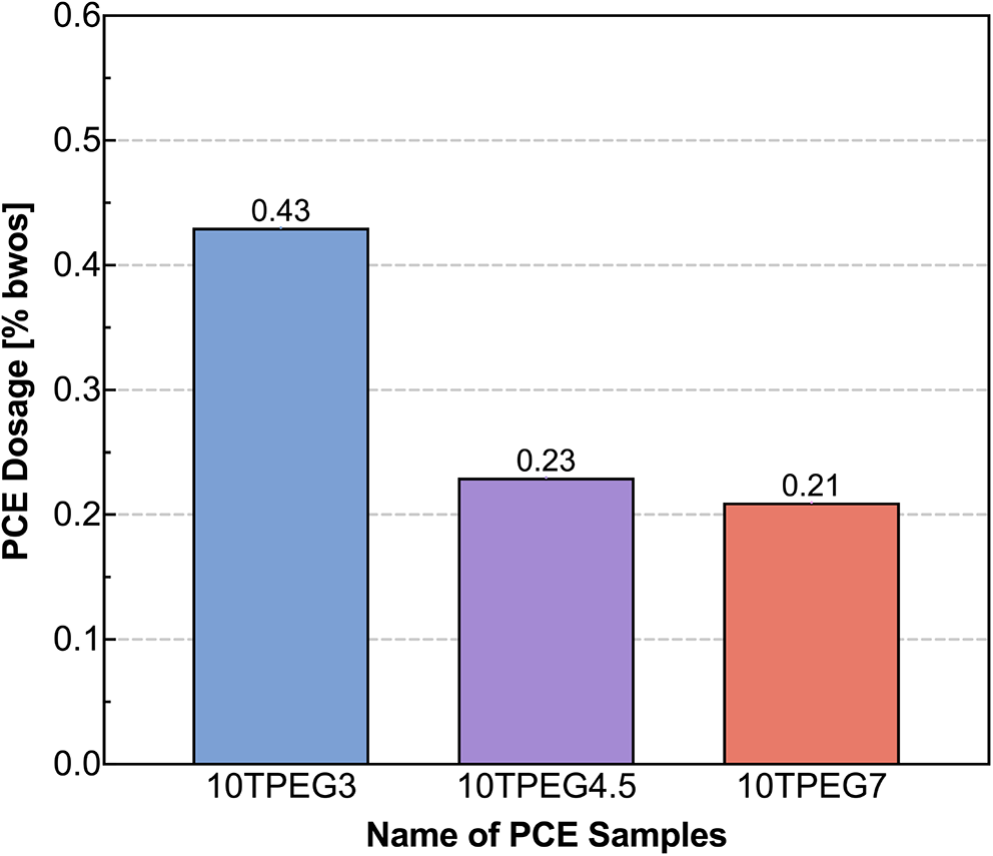
PCE dosage needed to achieve a spread flow of 26 ± 0.5 cm in the mini-slump test on AAS pastes (w/s = 0.6; reference spread flow: 18.3 cm).

### 3.3 Interaction Modes between PCE Polymers and the AAS Paste System

#### 3.3.1 ζ-Potential

Initially, the surface of the slag particle is positively charged due to the cation-rich ions (e.g., Ca^2+^ and/or Mg^2+^) that dominate the early NaOH-activated slag pore solution, resulting in ∼+3.00 to +3.25 mV ζ-potential, as shown in Figure 5. However, as PCE samples are adsorbed onto the slag particles, the ζ-potential subsequently shifts toward neutrality with increasing PCE dosage via titration. Such continuous reduction in ζ-potential indicates the negatively charged PCE molecules are successfully adsorbed on the positively charged slag particles, and the ζ-potential approaches a plateau. Among the samples, 10TPEG7 exhibits the lowest ζ-potential across all dosages and the greatest Δζ-potential, indicating higher adsorption and greater surface charge compensation. This phenomenon is likely related to the greater anionic charge density of 10TPEG7, as the increased number of COO^–^ groups may promote stronger adsorption and surface charge compensation on Ca-rich sites. Thus, as 10TPEG7 adsorbs more densely onto the slag particles, the positive surface charge becomes partially shielded. Moreover, such stronger adsorption can provide superior steric stabilization, as better surface coverage can provide greater dispersion. However, the mechanism of such dispersing effectiveness is not purely electrostatic but is dominated by steric hindrance.^36^ In contrast, the slower decay of ζ-potential from 10TPEG3 and 10TPEG4.5 suggests lower adsorption affinity and lower surface coverage. To confirm the higher adsorption from 10TPEG7, a TOC measurement was performed.

**Figure 5:**
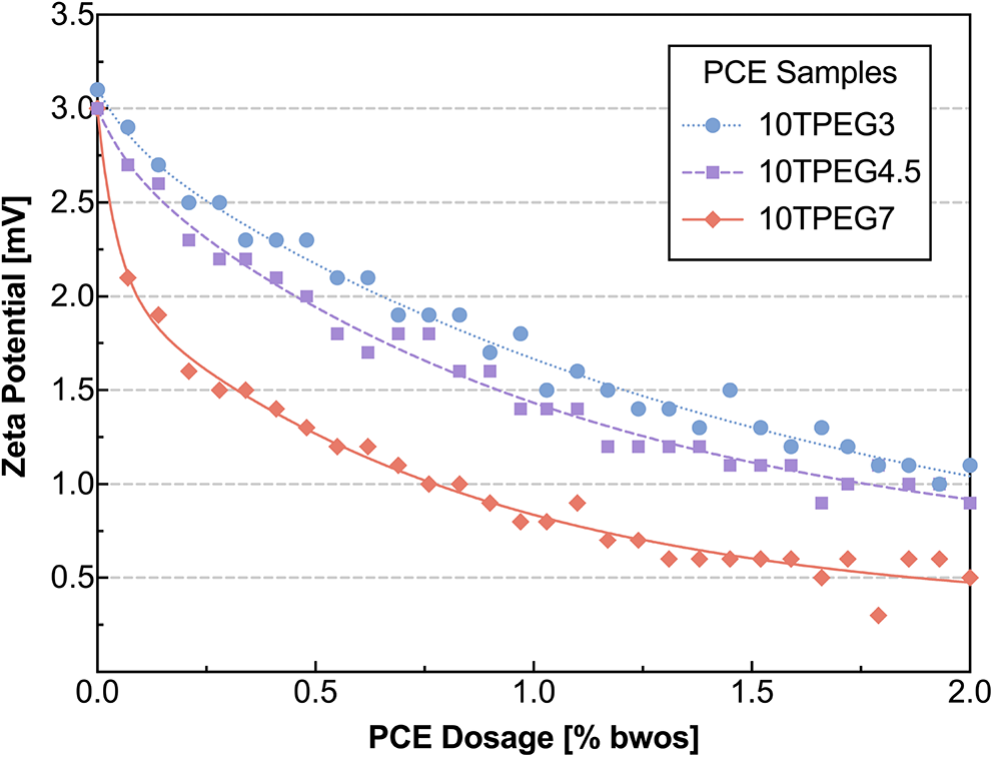
ζ-potential of AAS paste during the addition of 2% bwos to three TPEG-based PCE solutions (w/s = 0.6).

#### 3.3.2 Adsorption Isotherm

It is shown that increasing the PCE dosage progressively reduces the surface charge of slag particles (Figure 5) and increases the adsorbed amount of PCE onto slag particles (Figure 6). However, Ca^2+^ complexation may play a significant role since an incomplete correspondence between the ζ-potential plateau and adsorption saturation was observed. The Ca^2+^ chelation by PCEs becomes even more critical when the polymers are in a high-pH, high-ionic-strength environment and when the Ca^2+^ concentration is limited, such as in the AAS pore solution. As illustrated in Figure 6, the adsorption of all PCEs onto AAS pastes was found to be proportional to their dosage, exhibiting Langmuir isotherms until saturation was reached. Among the samples, 10TPEG7 exhibits the highest adsorption capacity (∼2.85 mg/g slag), followed by 10TPEG4.5 and 10TPEG3. Such an ascending adsorption trend correlates with the anionic charge density of the polymers; a similar result was reported by Lei et al. for HPEG instead of TPEG.^36^ Such an adsorption trend also suggests the importance of the number of carboxylate groups (−COO^–^) available in the PCE structure, since these functional groups serve as anchoring sites that interact with Ca^2+^-rich surface regions. As a result, 10TPEG7 exhibits the strongest Ca^2+^ complexation ability, possibly promoting the formation of more stable adsorption complexes and a greater reduction in ζ- potential, ultimately leading to a denser PCE adsorption layer.

**Figure 6:**
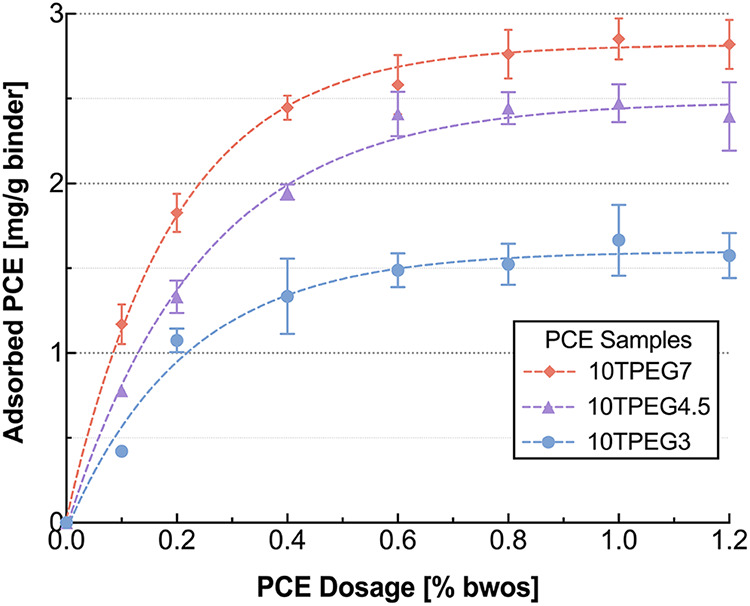
Adsorption isotherm of a series of TPEG PCEs (*n*_EO_ = 10) possessing different anionic charge densities in AAS pastes (w/s = 0.6).

A possibly more compact polymer adsorption layer can induce stronger steric stabilization, reduce particle flocculation, and improve dispersion. However, this stabilization may delay the initial hydration kinetics and prevent localized precipitation. Consequently, it may delay the nucleation and growth of hydration products such as C-(A)-S-H. Thus, the higher adsorption capacity and stronger surface binding of 10TPEG7 may prolong the setting time in the AAS system. This relationship will be further discussed in Section 3.4.

### 3.4 Setting Time of AAS Pastes with PCEs

Setting time experiments were conducted to ascertain whether the PCEs exert a retardation effect on the AAS binder system, as several studies had previously reported that the addition of PCEs demonstrated such an effect in cement-based systems.^44^ Here, the dosages of PCE are all fixed at 0.3% bwos to prevent confounding effects caused by different dosages. As illustrated in Figure 7, the incorporation of PCEs extended both the initial and final setting times relative to the reference, thereby indicating a retardation effect of PCEs on AAS pastes. Such a retarding behavior becomes more pronounced as the PCEs’ anionicity increases (10TPEG7 > 10TPEG4.5 > 10TPEG3). Moreover, it is found that the degree of retardation is correlated with PCE adsorption capacity, with higher adsorbed amounts (e.g., 10TPEG7 ∼ 2.85 mg/g slag) corresponding to longer setting times, as shown in Table 4. Although TOC adsorption and setting time measurements were conducted at different w/s ratios (0.6 and 0.4, respectively), the comparison is intended to highlight overall adsorption–retardation trends rather than direct quantitative equivalence. The ζ-potential results (Figure 5) imply stronger adsorption from high-anionicity PCE due to its stronger Ca^2+^ complexation ability (Figure 3). With stronger adsorption, PCEs with higher anionicity may then exhibit greater surface coverage, ultimately resulting in better dispersion. However, the retardation may be associated with stronger adsorption and effective dispersion. Similar PCE-induced retardation phenomena have been observed in OPC and NaOH-activated systems.^30,45^ However, the stronger correlation between adsorption and retardation in AAS pastes highlights the importance of adsorption behavior under calcium-deficient environments. With this, the subsequent section on the calorimetric analysis (Section 3.5) will further examine how polymer adsorption modifies hydration kinetics.

**Figure 7:**
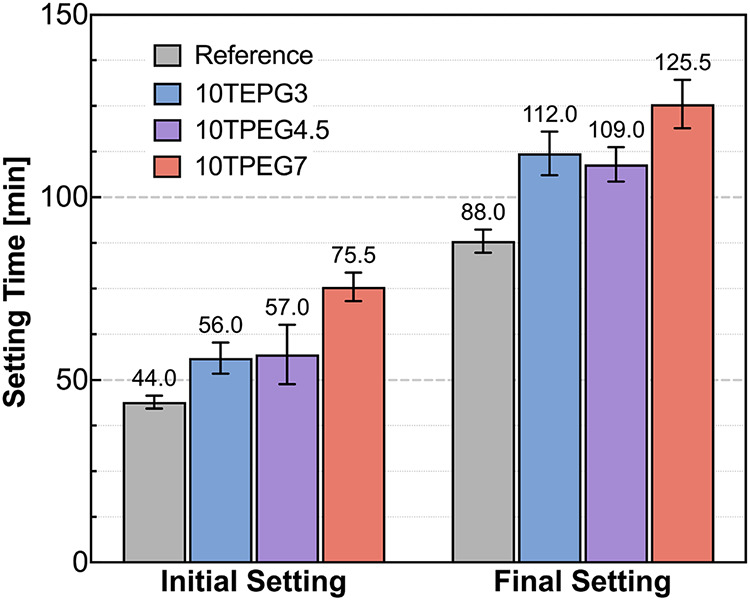
Setting time of AAS pastes with and without PCEs (w/s = 0.4); fixed PCE dosages at 0.3% bwob.

**Table 4:** Increase in Setting Time Relative to PCE-Free AAS Paste and PCE Adsorption on AAS Paste Measured by TOC

PCE samples	Initial setting time [min]	Final setting time [min]	Δ*t* ^a^ [min]	Saturated adsorbed amount of PCE^b^ [mg/g slag]
10TPEG3	+27.3%	+27.3%	+27.3%	1.58
10TPEG4.5	+29.5%	+23.9%	+18.2%	2.47
10TPEG7	+71.6%	+42.6%	+13.6%	2.85

a Δ*t* [min] = initial setting time [min] – final setting time [min].

b The experimental conditions of TOC measurement were w/s = 0.6, and the experimental conditions of setting time measurement were w/s = 0.4.

### 3.5 Heat Flow Calorimetry of AAS

Based on the isothermal calorimetry measurements, as shown in Figures 8 and 9, three major scenarios can be observed: (a) slag without NaOH; (b) slag activated with NaOH; and (c) NaOH-activated slag containing PCEs. In the first case, no discernible heat was observed even after 48 h, indicating a negligible hydration reaction under these conditions. On the other hand, the incorporation of NaOH markedly accelerates the hydration process, giving rise to a pronounced initial dissolution peak at approximately 3–4 h. This peak is attributed to the rapid breakdown of the aluminosilicate glass network. Subsequently, precipitation and polymerization lead to the formation of C-(A)-S-H gel, resulting in a gradual decline in intensity. These observations are consistent with those of previous studies.^18^

**Figure 8:**
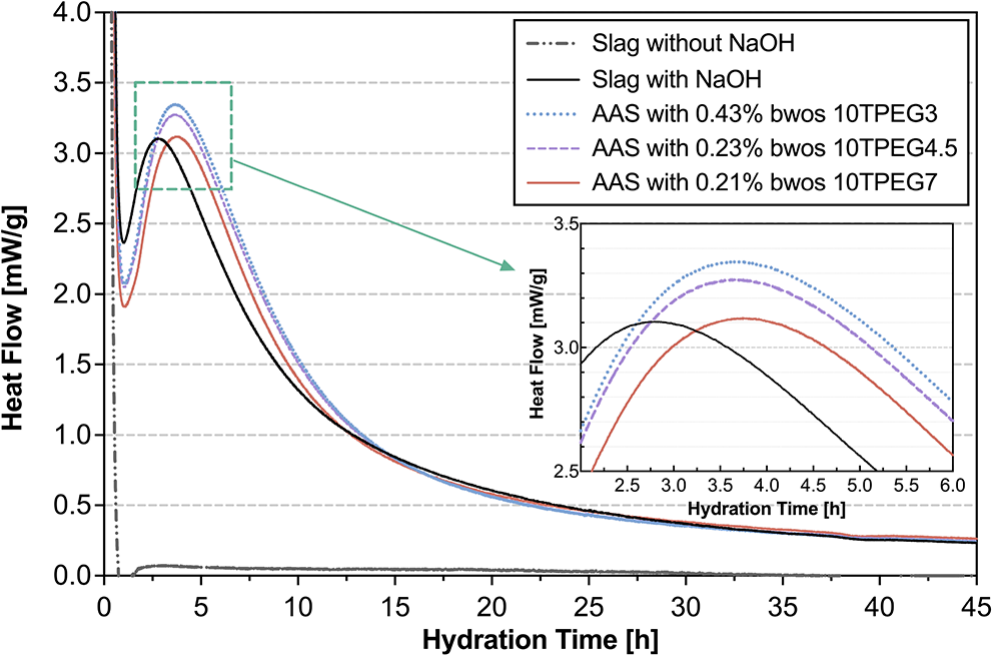
Heat flow evolution curves of slag paste in the absence/presence of activator (NaOH) and AAS pastes with PCE possessing different anionicities (w/s = 0.6).

**Figure 9:**
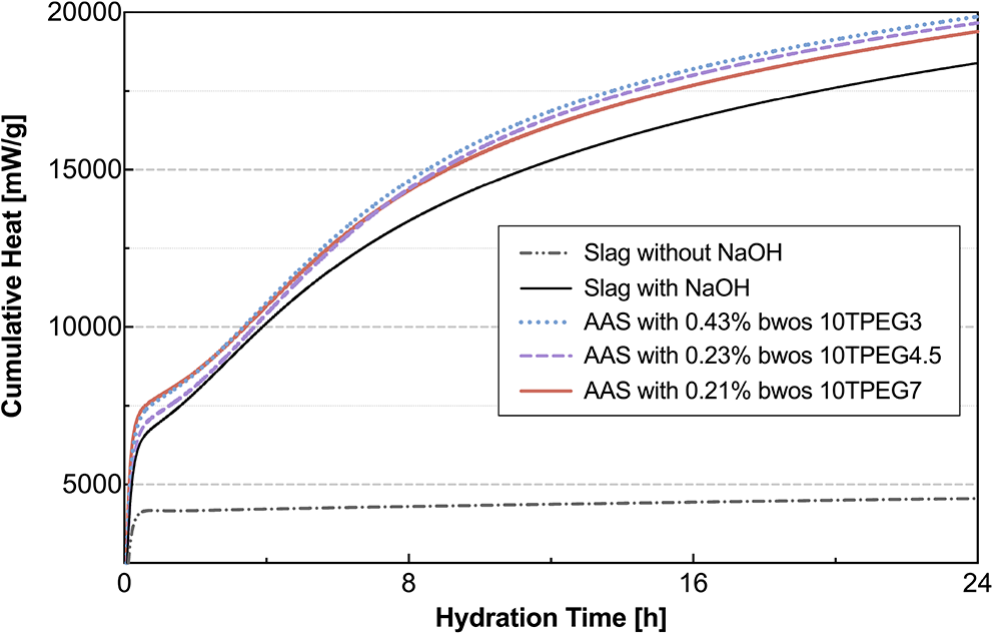
Heat cumulative curves of slag paste in the absence/presence of activator (NaOH) and AAS pastes with PCE possessing different anionicities (w/s = 0.6).

In the circumstances of the third scenario, it is shown that the incorporation of PCEs into the NaOH-activated systems does not fundamentally change the shape of the calorimetric curves but results in systematic variations in peak intensities and times of occurrence. The main peak intensity decreases and shifts slightly to the right as anionicity increases (10TPEG7 < 10TPEG4.5 < 10TPEG3). Among them, 10TPEG7 shows a lower intensity and a slightly delayed peak, indicating that this PCE structure slightly retards the early hydration kinetics. This behavior is in line with its stronger adsorption affinity and Ca^2+^ complexation capacity (Figure 3), as well as its pronounced retarding effect on the setting time (Figure 7). Moreover, such retarding behavior may therefore be attributed to greater adsorption, thus better dispersion, and ultimately modification of the early-stage kinetics of the AAS system.

In the case of cumulative heat (Figure 9), PCE-containing samples exhibit a slightly higher total heat release, despite early suppression. This indicates that the presence of PCEs delays AAS hydration only slightly, as shown in the setting time experiment (Figure 7) but does not inhibit it (Figure 9). This suggests that PCEs mainly modify early-stage reaction kinetics rather than reducing the total hydration extent.

### 3.6 Impacts of PCE on the Early Strength of AAS Mortars

In addition to the influence of PCEs on setting time and early hydration kinetics (Section 3.5), the presence of superplasticizers is expected to affect the development of compressive strength of AAS mortars, as modifications to early-age hydration can alter the C-(A)-S-H formation. At the same time, PCE-induced particle dispersion may enhance hydration efficiency and contribute positively to strength development at both early and later ages. Thus, three PCE samples were applied in the mortar compressive strength test, and all samples were prepared under comparable air-content conditions (mortar density of 2.59 ± 0.05 g/cm^3^) via the addition of defoamer and under controlled fluidity (a spread flow of 22 ± 0.5 cm). This approach was adopted in view of the observed impact of PCEs on strength in the OPC system, despite maintaining a fixed w/c ratio.^46^

Compressive strength was measured at 8 h, 16 h, 1 d, 3 d, 7 d, and 28 d to capture both early- and late-age performance. The PCE dosages required to achieve the targeted workability are presented in Figure 10. The mortar dosage trend is consistent with that observed in the mini-slump test (Figure 4), confirming that 10TPEG7 requires a lower dosage to attain the desired fluidity, particularly compared to 10TPEG3.

**Figure 10:**
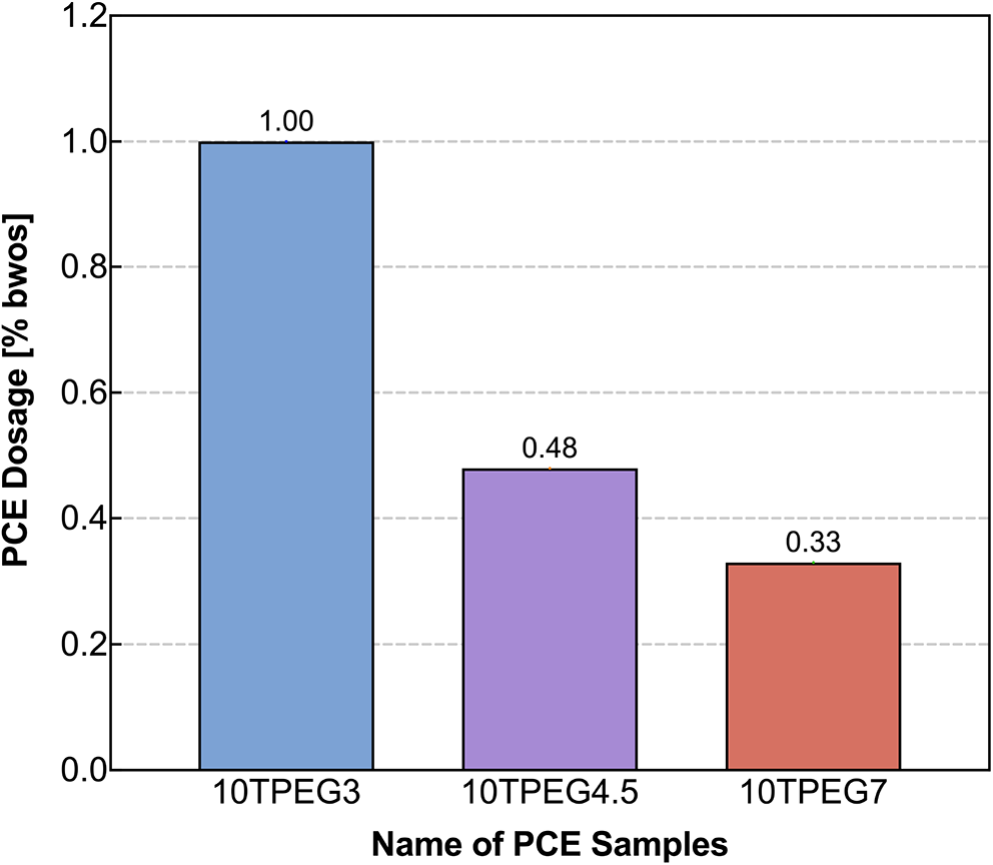
Dosage of PCEs to achieve a mortar paste spread flow of 22 ± 0.5 cm in the mortar test (w/s = 0.5; reference spread flow = 17.5 cm).

All PCE-containing mortars exhibit higher compressive strength than the reference sample, suggesting a positive impact on hydration efficiency from the dispersion of PCEs, as shown in Figure 11. However, distinct differences are observed among the three admixtures at early ages. Interestingly, mortars containing 10TPEG3 exhibit a lower compressive strength at early curing ages (8 and 16 h) than 10TPEG4.5 and 10TPEG7, despite exhibiting a higher heat evolution peak in isothermal calorimetry. This discrepancy highlights that the magnitude of the heat flow peak does not directly correlate to mechanical performance, as isothermal calorimetry primarily reflects the hydration kinetics rather than the efficiency of C-(A)-S-H gel formation.

**Figure 11:**
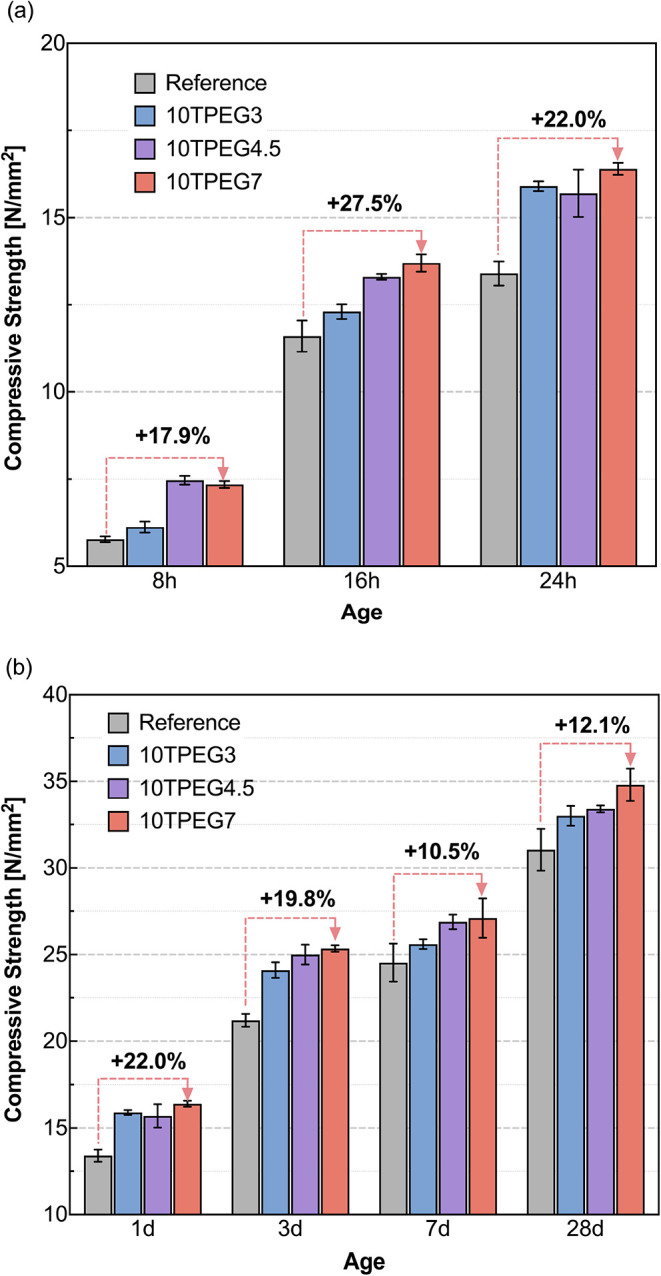
Compressive strength of AAS mortar (w/s = 0.5) after curing for (a) 8, 16, and 24 h; (b) 1, 3, 7, and 28 d.

One possible explanation is that the higher dosage of 10TPEG3 required to achieve the target workability may lead to rapid, unstable early hydration, resulting in a likely heterogeneous distribution of hydration product and a coarser microstructure. In contrast, the slower, more gradual hydration reactions in 10TPEG4.5 and 10TPEG7 favor the formation of a denser, more interconnected gel matrix. In particular, 10TPEG7 exhibits high anionicity, inducing the most pronounced delay in early hydration (longer setting time), which may facilitate the gradual formation of a denser C-(A)-S-H network due to the possible prolongation of dissolution, ultimately producing slightly improved early- and long-term strength. Nevertheless, the compressive strength of all PCE samples in the later ages is rather similar, indicating that the specimen containing 10TPEG3 still gains strength despite lower adsorption affinity and weaker dispersibility during the fresh state.

PCE-induced retardation has been widely attributed to surface coverage^47^ and calcium complexation mechanisms.^48^ In the present study, the strength behavior observed for 10TPEG3 can be explained by a series of phenomena originally caused by a weaker adsorption affinity. Even with a relatively high dosage (1% bwos) of 10TPEG3, weaker particle stabilization is expected because of the nature of the polymer structure. Consequently, earlier precipitation may contribute to the high kinetic heat and a lower compressive strength.

### 3.7 Hydrates of AAS with PCEs—XRD

To determine the hydration products of GGBFS activated with NaOH, AAS paste samples cured for 24 h were immersed in ethanol to stop hydration and then analyzed by XRD. As illustrated in Figure 12a, the dominant reaction product is calcium-alumino-silicate-hydrate (C-(A)-S-H),^49^ rather than calcium-silicate-hydrate (C-S-H), which is consistent with the incorporation of Al into the silicate network during slag activation. Furthermore, all samples exhibit the åkermanite peak, indicating the presence of unreacted or residual slag that still needs to undergo dissolution at 1 d. Moreover, the appearance of the åkermanite peak suggests the potential for strength development, which was indeed observed in all samples as the dissolution process continued. Additionally, magnesium aluminum hydroxide (Mg_4_A_l2_(OH)_14_·3H_2_O), also known as hydrotalcite, was identified at ∼10.9° and ∼26.1°. Hydrotalcite is a layered double hydroxide (LDH) compound based on brucite-type layers with interlayer water and carbonate ions, which can be replaced by other anions, resulting in alterations to the cell parameter.^50^ The presence of hydrotalcite indicates that the alkali solution effectively activated the slag particles, as hydrotalcite typically precipitates when Al^3+^ and Mg^2+^ ions are dissolved from slag particles in an alkaline environment.

**Figure 12:**
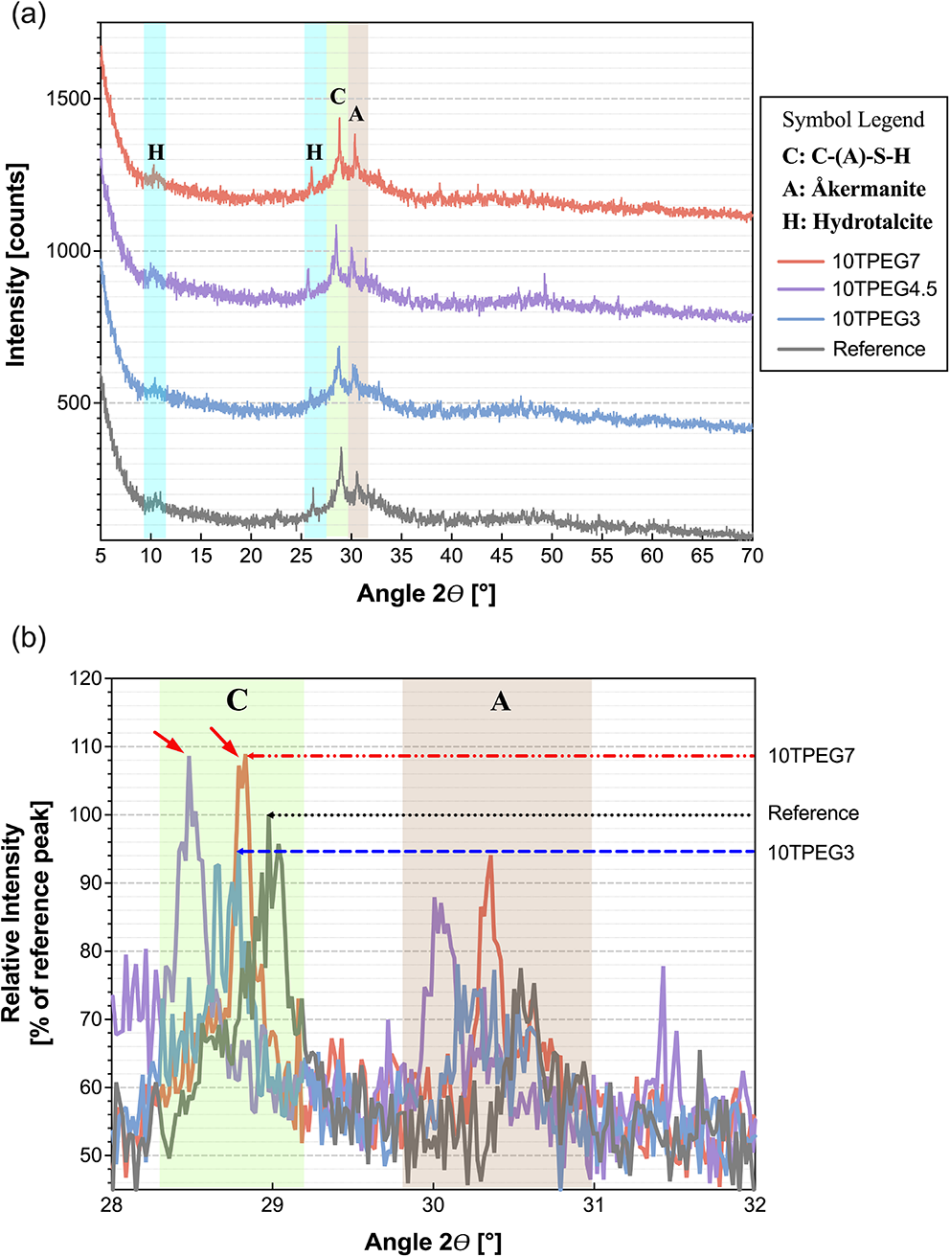
XRD patterns of NaOH-activated AAS pastes after 1 d of curing: (a) full diffraction patterns (5–70° 2θ) and (b) enlarged view of the C-(A)-S-H region showing relative intensity normalized to the reference peak.

Comparison of the XRD patterns from three different PCE samples reveals that 10TPEG7 and 10TPEG4.5 display a broader amorphous region associated with C-(A)-S-H as compared to 10TPEG3. The intensity from this amorphous region provides only limited semiquantitative information and reflects gel-related scattering contributions, meaning that it is not a direct measure of the absolute amount of hydrate formed. Nevertheless, it can be observed that the low-anionicity PCE (10TPEG3) presents a slightly lower relative intensity in the C-(A)-S-H region compared with the reference sample, whereas medium- and high-anionicity PCEs (10TPEG4.5 and 10TPEG7) show increased relative intensity when the diffraction intensity is scaled relative to the reference peak, as shown in Figure 12b. However, the result of XRD should not be overinterpreted since it is not suitable for direct quantification of amorphous hydration products such as C-(A)-S-H without additional quantitative methods. Therefore, the current XRD result can only provide qualitative evidence for possible differences in hydration-product evolution.

### 3.8 Mechanistic Discussion

In this study, all 10TPEG-based PCEs improve the dispersion of AAS particles and enhance the hydration efficiency: two key observations that warrant further investigation. First of all, although the addition of PCEs can delay the setting time and slightly reduce the isothermal calorimetry heat peak, they still provide better mortar strength development than that of the reference specimen. Second, the most anionic and dosage-advanced polymer, 10TPEG7, despite generating delayed hydration and extending the setting time, consistently achieved high strength across all ages, especially compared to 10TPEG3 at early curing ages (8 and 16 h). These apparently contradictory findings highlight the complex relationship among the polymer structure, ion complexation, and hydration dynamics.

In the absence of PCEs, the AAS mortar exhibits lower compressive strength at both early- and late-curing ages. As the NaOH activation system’s hydration process is rather exothermic and vigorous, researchers observe greater heat evolution than in other alkali-activated systems, such as Na_2_CO_3_ and Na_2_SiO_3_.^40,51,52^ Besides the possible flash set when the water-to-binder ratio is too low, other drawbacks of NaOH activation include a less homogeneous microstructure.^18^ Although the hydration of NaOH-activated AAS is initially driven by a dissolution–precipitation mechanism, at later stages the reaction continues by a solid-state mechanism.^50^ Gebregziabiher et al. observed that the surface of slag grains forms a thin reaction shell during the early hydration of the NaOH-activated system under the scope of BSE-SEM.^53^ Similarly, Haha et al. also reported that “rims” of hydrates are formed around NaOH-activated slags; in contrast, no clear rims are found in the case of the waterglass-activated system,^18^ showing distinct characteristics of the NaOH-based system. Under such conditions, maintaining effective particle separation and ion transport may be important for sustaining hydration.

Such sustained hydration conditions may also influence the evolution and organization of hydration products. Additionally, recent multiscale investigations presented by Huang and Wang have shown that the formation of C-(A)-S-H in NaOH-activated slag proceeds from nonclassical nucleation toward increasingly ordered tobermorite-like configurations, which are associated with enhanced structural coherence of the gel network.^54^ This suggests that a more effective dissolution of slag particles may favor the development of a denser C-(A)-S-H structure and that the resulting hydration products can lead to higher compressive strength.

In this context, the enhanced compressive strength of AAS mortar can plausibly be explained by the addition of PCE samples, which favor the dissolution of AAS particles by dispersing them. The existence of a dispersant can not only stabilize the slag particles but also possibly delay local precipitation. This modulation of rim evolution could make the ion transport more favorable, reducing diffusion limitation. Based on this mechanism, the presence of PCEs can either (a) improve accessible surface exposure of unreacted slag particles and/or (b) promote a longer dissolution process at the early hydration stage (curing age > 4 h). This interpretation is partially consistent with Schönlein et al.’s study, where they observed that the presence of PCEs can generate a core–shell morphology onto synthetic C-S-H; this rim delays the conversion to nanofoils for several hours by stabilizing the metastable globules.^55^ This may explain the apparent contradiction that, despite the paste containing PCEs having a longer setting time, it can still exhibit greater compressive strength than the reference sample.

Therefore, a well-dispersed stabilizer, such as 10TPEG7, is beneficial for hydration, as its stronger Ca^2+^ chelation, driven by its high anionicity, can promote higher adsorption and more effective dispersion, as shown in Figure 13a. Although it initially retards hydration, as shown in longer setting time (Figure 7) and lower heat flow peak (Figure 8), this high-anionicity PCE structure may provide effective particle dispersion while sustaining dissolution conditions. This prolonged dissolution process may favor the growth of C-(A)-S-H, potentially resulting in a stronger matrix at early curing ages, as illustrated in Figure 13b.

**Figure 13:**
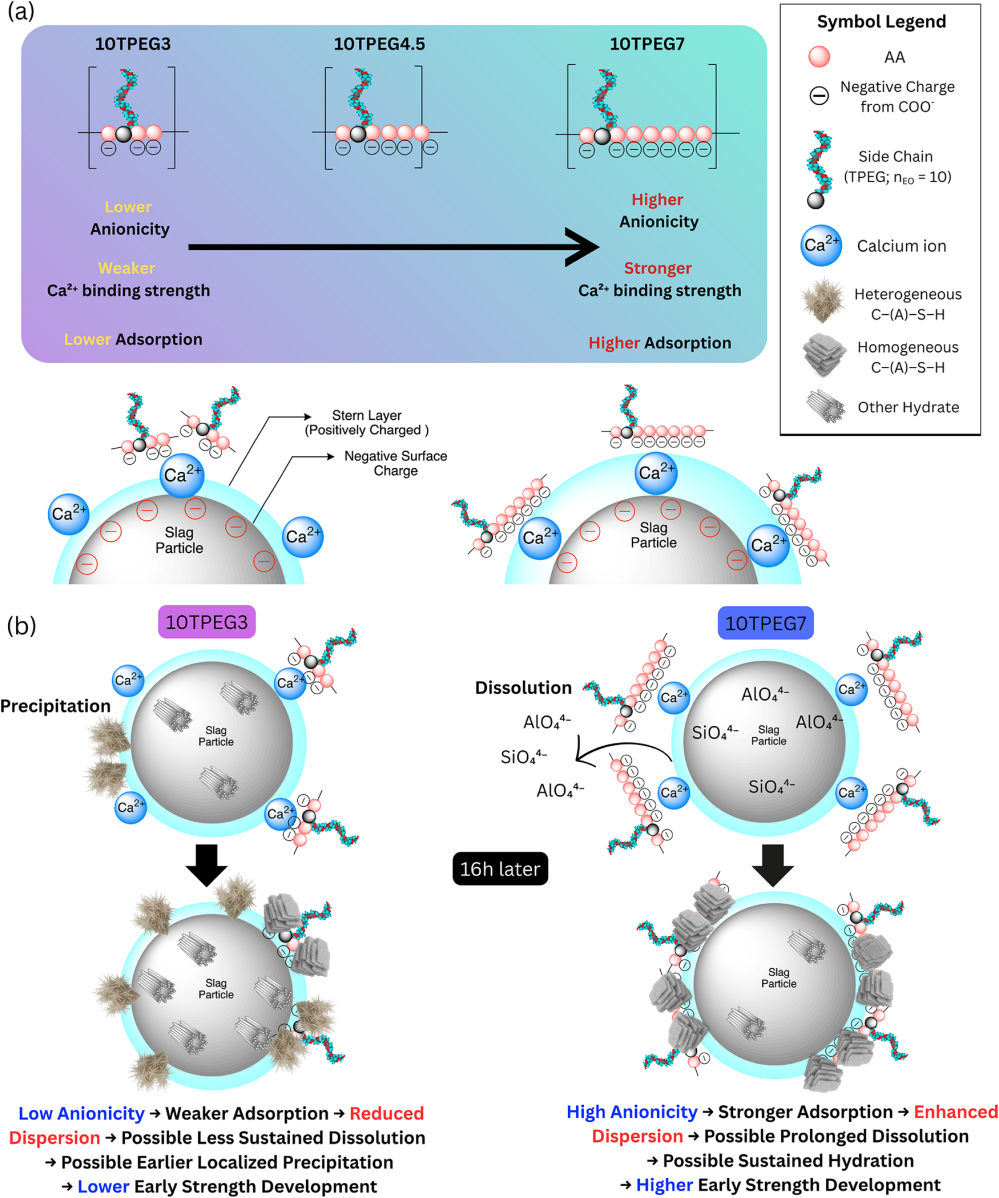
Mechanistic illustration of possible PCE–AAS interactions as influenced by polymer anionicity. (a) Increasing anionic charge density enhances polymer adsorption through stronger Ca^2+^ chelation and (b) enhanced dispersion in high-anionicity PCEs (10TPEG7) may contribute to prolonged dissolution and sustained hydration progression, whereas low-anionicity PCEs (10TPEG3) may favor less sustained dissolution behavior and weaker early-age strength development.

In contrast to 10TPEG3, it requires a higher dosage to achieve the desired workability due to insufficient adsorption, resulting in less effective stabilization. This not only causes poorer particle separation but also may interfere with early gel polymerization and trigger localized precipitation. This could then produce a less homogeneous microstructure, leading to worse early strength development. Such behavior may also be consistent with the possible differences observed in the XRD pattern (Figure 12b). Although the low-anionicity PCE shows a lower relative intensity in both the C-(A)-S-H and åkermanite-related XRD region, it still exhibits higher compressive strength than the reference. This indicates that strength enhancement is still possible with improved particle stabilization and better particle packing; one of the drawbacks is that 10TPEG3 requires a much higher dosage to achieve comparable workability.

Nevertheless, further studies should be conducted, since this work can support the presence of optimized PCE structures (e.g., 10TPEG7) in such an alkaline environment, which can not only enhance dispersing effectiveness but may also contribute to a longer setting time and higher compressive strength. It could be interesting to discover whether or not the PCE dispersants can result in the development of a complex microstructure consisting of fractal assemblies of nanometer-sized discs,^49^ leading to higher fractal dimensions and closer disc packing, ultimately forming a more refined and dense long-term matrix. Moreover, the conformational effects should also be considered, since highly alkaline environments may influence polymer conformation and adsorption behavior.

## 4 Conclusions

This study aims to investigate the impact of the anionicity of 10TPEG-based PCE on early hydration kinetics and setting time behavior in the NaOH-activated slag system. The combined analyses of anionic charge measurement, dispersion performance, adsorption capacity, ζ-potential, calorimetry, setting time, compressive strength, and XRD provide a comprehensive molecular-to-macro understanding of PCE–AAS interactions.

All 10TPEG-based copolymers were observed to improve the dispersion between AAS particles and slightly retard early hydration kinetics as observed by calorimetry results; however, neither early- nor late-strength development was compromised as compared to the reference sample. It was demonstrated that 10TPEG7 exhibited higher adsorption and remarkable dispersing ability. The trade-off of this effect is that 10TPEG7 delivered a longer setting time and lower kinetic heat. Nevertheless, both early- and late-mechanical properties of 10TPEG7 still outperformed the other two PCE samples. A possible explanation is that better particle stabilization may not only delay the local precipitation (longer setting time) but also possibly prolong dissolution. As the dissolution process extends, the microstructure of C-(A)-S-H could be more homogeneous since the hydration of the NaOH-activated system is known to be highly reactive. If hydration progression is sustained, then the compressive strength is likely to be higher.

Although this hypothesis should be further validated, the overall findings reveal a structure–property relationship linking the molecular design of PCEs with the workability, early hydration, and mechanical behavior of NaOH-activated AAS. The anionicity is a key structural factor that influences the (i) workability, (ii) setting time and isothermal heat, and (iii) potential characteristics of early hydrates, particularly C-(A)-S-H. In summary, this study provides molecular-level guidance for designing next-generation PCEs for such a green binder system, particularly in alkali-activated systems, and is expected to advance the development of high-performance, low-carbon binder technologies.
